# VHL restoration in clear cell renal cell carcinoma improves NK cell infiltration and function

**DOI:** 10.1007/s00262-025-04132-x

**Published:** 2025-07-30

**Authors:** Le Tong, Apple Hui Min Tay, Weiyingqi Cui, Yaxuan Liu, Yanhong Su, Jiawen Lyu, Leila Hoedemakers, Ying Yang, Monika Ehnman, Barbara Seliger, Par Nordlund, Felix Haglund de Flon, Shi Yong Neo, Andreas Lundqvist

**Affiliations:** 1 Department of Oncology-Pathology, Karolinska Institutet, J6:20 BioClinicum, Akademiska straket 1, Solna, 17164 Stockholm, Sweden; 2 Key Laboratory of Quantitative Synthetic Biology, Shenzhen Institute of Synthetic Biology, Shenzhen Institute of Advanced Technology, Chinese Academy of Sciences, Shenzhen, China; 3 School of Biological Science, Nanyang Technological University, Singapore, Republic of Singapore; 4 Faculty of Health Sciences Brandenburg, Institute of Translational Immunology, Medical School “Theodor Fontane”, Brandenburg an Der Havel, Germany; 5 Center of Translational Medicine, Medical School “Theodor Fontane”, Brandenburg an Der Havel, Germany; 6 Medical Faculty, Martin Luther University of Halle-Wittenberg, Halle (Saale), Germany; 7 Agency for Science, Technology and Research (A*STAR), Singapore Immunology Network, 8A Biomedical Grove, Singapore, 138648 Republic of Singapore

**Keywords:** VHL, Clear cell renal cell carcinoma, NK cells, Infiltration, Spheroid, Organoid

## Abstract

**Background:**

The von Hippel–Lindau (VHL) gene is frequently mutated in clear cell renal cell carcinoma (ccRCC) which results in stabilization of hypoxia-inducible factor (HIF). Despite the well-known immunosuppressive effect of HIF, ccRCC is considered an immunogenic tumor with high lymphocyte infiltration. Since NK cells have a prognostic value in ccRCC patients, it is important to understand how VHL mutations affect NK cell activity and anti-tumor immunity.

**Methods:**

Tumor spheroids were generated from parental 786-O (VHL-mutated) and 786-O-pVHL (VHL-restored) ccRCC cell lines. Tumor phenotypes, proteome, and secretome were analyzed by flow cytometry, mass spectrometry, and Luminex assays, respectively. Quantitative proteomics analysis and quantitative gene ontology enrichment were used to correlate protein expression changes to ccRCC progression and immunosuppressive pathways. NK cell infiltration, activation, and cytotoxicity were assessed in co-cultures of ccRCC spheroids with NK cells from healthy donors using real-time imaging, immunostaining, and flow cytometry, respectively.

**Results:**

VHL-mutated tumor spheroids were significantly less infiltrated by NK cells compared with VHL-restored tumor spheroids. pVHL-infiltrating NK cells showed an activated phenotype along with the ability to reduce tumor spheroid size. Proteomic analysis revealed that VHL-restored tumors express reduced levels of proteins associated with ccRCC progression and immunosuppression, including components of MHC class I processing and PD-1 signaling. Furthermore, VHL-restored tumors exhibited decreased levels of hypoxia-related and pro-tumoral cytokines, such as GROα, IL-8, IL-10, TRAIL, VEGF, and SCF. Within 768-O tumor spheroids, NK cells displayed a higher degree of hypoxia and expression of HIF1α, and inhibition of HIF1α resulted in higher NK cell infiltration into 786-O spheroids. Similarly, inhibition of the VHL-target gene, HIF2α, in 786-O spheroids resulted in increased NK cell infiltration.

**Conclusions:**

VHL mutant tumors are less infiltrated by NK cells due to immunosuppressive pathways driven by HIF stabilization. Restoration of VHL reprograms the tumor microenvironment, reducing ccRCC progression and immunosuppressive signaling while enhancing NK cell infiltration and activation. Inhibition of HIFα improves NK cell infiltration into VHL mutant tumors. Therefore, inhibition of HIFα should be explored as a therapeutic strategy in ccRCC to improve NK cell anti-tumor efficacy against VHL-mutated tumors.

**Supplementary Information:**

The online version contains supplementary material available at 10.1007/s00262-025-04132-x.

## Background

Renal cell carcinoma (RCC) is a common malignancy of the kidney that originates from the tubular epithelial cells [1]. It accounts for approximately 3% of all adult malignancies and has the highest mortality rate among urologic cancers [2]. RCC is classified into four main subtypes, where clear cell RCC (ccRCC) comprises 75% of all RCC cases [3]. One of the hallmarks of ccRCC is the mutation in the von Hippel–Lindau (VHL) gene [4], which occurs in up to 90% of ccRCC cases [3]. VHL is a tumor suppressor and E3 ubiquitin ligase that degrades hypoxia-inducible factor (HIF) under normoxic conditions. Inactivation of VHL leads to oxygen-independent activation and stabilization of HIF, creating a state of pseudohypoxia [5]. This results in activation of various tumor-associated pathways, including angiogenesis, altered metabolism, and cell proliferation, which contribute to ccRCC progression [3].

ccRCC is an immunogenic tumor defined by high infiltration of CD8 cytotoxic T cells and natural killer (NK) cells [6, 7]. Unlike other immunogenic tumors including melanoma and non-small cell lung cancer, the presence of T cells has been associated with poor prognosis in ccRCC [8, 9]. In contrast, the presence of NK cells within the tumor microenvironment (TME) is associated with a better survival rate [8, 10]. Therefore, it is important to understand the role of NK cells in ccRCC and their potential as a therapeutic target.

NK cells represent a key component of the innate immune system and play a critical role in the recognition and elimination of tumor cells. Despite the increasing interest to exploit the anti-tumor activity of NK cells for therapeutic purposes, there is a significant knowledge gap in how NK cell activity is governed in ccRCC. Notably, accumulation of HIF promotes immune evasion through suppression of T and NK cells [6, 7, 11]. This immunosuppression can be linked with the upregulation of immune checkpoint molecules such as programmed death-ligand 1 (PD-L1). As a result, ccRCC-infiltrating NK cells exhibit an exhausted phenotype characterized by decreased expression of activating receptors and cytokine production [6, 8, 12, 13].

In this study, we investigated the activity of NK cells in relation to the VHL status in ccRCC. Using quantitative proteomic analysis along with in vitro co-culture models, increased NK cell infiltration and activity was observed upon VHL restoration. Furthermore, inhibition of HIF1α or HIF2α in NK cells improves their infiltration into VHL-mutated tumors. As such, strategies to inhibit HIFα expression represent a valid approach to improve NK cell anti-tumor efficacy against VHL-mutated tumors.

## Materials and methods

### Cell culture

ccRCC cell lines 786-O (ATCC, Manassas, Virginia, USA) with innate VHL mutation and 786-O-pVHL (pVHL) with transfected VHL were maintained in RPMI-1640 supplemented with 10% heat-inactivated Fetal Bovine Serum (FBS) and 1% penicillin–streptomycin (PS, Thermo Fisher Scientific, Waltham, Massachusetts, USA) at 37 °C, 5% CO_2_ incubator. Stable GFP-expressing (> 90%) 786-O and pVHL cell lines were established using a third-generation lentiviral transduction system. The pLV-eGFP gift from Pantelis Tsoulfas (Addgene plasmid # 36083; http://n2t.net/addgene:36083; RRID: Addgene_36083) [14] was co-transfected with plasmids expressing the virus coat and assembly proteins (REV, RRE, and VSVG) into HEK293T cells at 70–80% confluency using Lipofectamine 3000 (Life Technologies, Carlsbad, California, USA). After 24, 48, and 72 h, the conditioned media were collected, filtered through 0.45 μm low-protein binding membranes (Sarstedt, Nümbrecht, Germany) and concentrated at 3200RCF for 15 min. Prior to infection, 786-O and 786-O-pVHL cells were seeded in T75 flasks and allowed to reach 70–80% confluency. The following day, the spent medium was removed and replaced with 2.5 ml of fresh media, 1 ml of medium containing lentivirus particles, and polybrene (Santa Cruz Biotechnology, Dallas, Texas, USA) at a final concentration of 8 μg/ml. After 16 h, the medium was discarded and substituted with fresh cell culture medium. GFP-positive cells were then sorted with a FACSAria Fusion (BD Biosciences).

Tumor spheroids were prepared by seeding 1 × 10^4^ cells per well in a 96-well ultra-low attachment plate and cultured for 5 days. Peripheral blood mononuclear cells (PBMCs) were isolated from healthy blood donors’ buffy coat by Ficoll density gradient centrifugation (Cytiva, Marlborough, Massachusetts, USA). NK cells were isolated from PBMCs using MACS MicroBead Human NK cell isolation kit (Miltenyi Biotec, Bergisch Gladbach, Germany) resulting in a purity of > 95% NK cells (Fig. S1A). Isolated NK cells were cultured for 2 days in X-VIVO 20 (Lonza, Basel, Switzerland) 10% heat-inactivated human AB serum (Karolinska University Hospital) and 1% PS supplemented with 300 IU/mL interleukin 15 (IL-15, PeproTech, Cranbury, New Jersey, USA). On day 5 of tumor spheroid culture, 3 × 10^4^ NK cells were added to tumor spheroids. Following 2 days of co-culture, spheroids were harvested and washed those in Dulbecco′s phosphate-buffered saline (DPBS, Thermo Fisher Scientific) prior to TrypLE™ (Thermo Fisher Scientific) digestion followed by flow cytometer (FC) analyses. Since expression of CD56 can vary on NK cells depending on different stimuli and various environmental signals within the tumor and be detected in tumor cells, CD45 was used to enumerate tumor-infiltrating NK cells. The majority (> 94%) of CD45-positive cells were also positive for CD56. Importantly, tumor cells were negative for CD45 (Fig. S1B). Where indicated, cell cultures were treated with anti-MHC class I (2.54 µg/ml W6/32, BioLegend, San Diego, California, USA), anti-ICAM-1 (20 µg/ml, BioCell, Irvine, CA, USA), anti-IL-10 (1 µg/ml BioLegend), anti-IL-8 (5 µg/ml BioLegend), the COX-2 inhibitor celecoxib (50 µM, MedChemExpress, Sollentuna, Sweden), the HIF1α inhibitor KC7F2 (20 µM, Sigma-Aldrich, Burlington, Massachusetts, USA), or the HIF2α inhibitor PT2385 (10 µM, MedChemExpress), respectively. For long-term culture to evaluate spheroid viability, tumor spheroids were cultured with NK cells for 5–7 days in the presence of 600 IU/ml IL-15.

### Flow cytometry analysis

Antibodies (Abs) used for flow cytometry (FC) are listed in Supplementary Table 1. Cell surface antibodies and live/dead (L/D) marker were incubated with samples at 4 °C for 20 min after washing twice with FC buffer containing 5% FBS in DPBS. Intracellular staining was performed using eBioscience™ (Waltham, Massachusetts, USA) fixation and permeabilization kit. Samples were washed and resuspended with FC buffer before acquiring on a NovoCyte (ACEA Bioscience, San Diego, CA, USA). To assess the hypoxia status, Image-iT™ green (irreversible) and red (reversible) hypoxia reagents (Thermo Fisher Scientific) were added directly to the tumor spheroids 1 day before FC acquisition. FlowJo software (Tree Star, Ashland, Oregon, USA) was used for data analysis. For degranulation and cytokine production assays, experimental setup was performed as previously published [15]. Briefly, NK cells were labeled with PE-Cy5.5 CD107a antibody for 30 min at 37 °C prior to incubation with tumor spheroids for another 30 min. Golgi stop and plug (BD Biosciences, Franklin Lakes, New Jersey, USA) were added according to manufacturer’s instructions. After 8 and 24 h of subsequent incubation, intracellular staining was performed for perforin, granzyme B, and interferon (IFN) y (Supplementary Table 1).

### Imaging

Brightfield and phase contrast images under 10 × objective were acquired every 4 h on IncuCyte S3 system (Essen BioScience, Ann Arbor, Michigan, USA). NK cells were labeled with CellTracker™ red CMTPX (Thermo Fisher Scientific) according to manufacturer's instructions for spheroid infiltration acquisition using red fluorescence. All spheroid formation and invasion analyses were performed using top hat segmentation with the IncuCyte software. Confocal imaging showing the hypoxic status using Image-iT™ hypoxia reagents of tumor spheroids was generated using the Opera Phenix™ High-Content Screening System (PerkinElmer, Waltham, Massachusetts, USA).

### LC–MS/MS

For protein extraction and quantification, tumor spheroids were washed with DPBS twice at 500 RCF for 4 min. Cell pellets were lysed with 2% SDS in 100 mM HEPES buffer (4-[2-hydroxyethyl]-1-piperazineethanesulfonic acid, VWR, Radnor, Pennsylvania, USA) with Pierce™ protease inhibitor tablet EDTA free (Thermo Fisher Scientific). To completely solubilize the proteins and break DNA, sonication was performed on ice. Supernatant was collected for protein quantification after centrifugation at 15 000 RCF for 10 min at 4 °C. Protein quantification was performed using Pierce™ BCA (bicinchoninic acid, Thermo Fisher Scientific) protein assay kit. Absorbances were read at 562 nm using the Versamax microplate reader (Molecular Devices, San Jose, California, USA). Quantified proteins were aliquoted and stored in −80 °C before proteomics analyses.

For proteomics sample preparation and LC–MS/MS analyses, filter-aided sample preparation [16, 17] and in-solution digestion [18] were performed as previously published. LysC (Wako, Waltham, Massachusetts, USA) and trypsin (Promega, Madison, Wisconsin, USA mass spectrometry grade) at a 1:50 enzyme to protein ratio were used at 0 and 3 h, respectively, and incubated at 37 °C for 16 h. Digested peptides were collected in TEAB (Triethylammonium bicarbonate buffer, Sigma) and labeled with isobaric Tandem Mass Tags, TMT-6plex according to manufacturer’s protocol (Thermo Fisher Scientific). The labeled samples were quenched using 1 M Tris pH 7.4 solution (Thermo Fisher Scientific) after labeling check. A high pH reverse phase Zorbax 300 Extend C-18 4.6 mm × 250 mm (Agilent Technologies, Santa Clara, California, USA) column, and liquid chromatography AKTA Micro (GE Healthcare, Chicago Illinois, USA) system was used for offline sample pre-fractionation. The fractions were concentrated into 20 fractions and dried by a centrifugal vacuum evaporator. Fractionated peptides were reconstituted in 0.1% formic acid (FA) for LC–MS/MS analysis in Dionex UltiMateTM 3000UPLC system coupled to a Q Exactive™ HF mass spectrometer as previously described (Thermo Fisher Scientific). Each sample was injected into EASY-Spray™ column (75 µm × 50 cm ID Acclaim™ PepMap™ RSLC C18, 3 µm, 100 A°; Thermo Fisher Scientific).

### Bioinformatics analyses

For proteomic analysis, raw output files from LC–MS/MS were processed using the Proteome Discoverer™ software version 2.1 (PD2.1). Protein identification was done by mapping against the UniProt KnowledgeBase (UniProtKB) Homo sapiens protein database (downloaded on Jan 13, 2017, including 42,105 sequence entries) and using SEQUEST-HT and Mascot 2.6.0 (Matrix Science, London, United Kingdom) search engines. MS precursor mass tolerance was set at 20 ppm, fragment mass tolerance set at 0.05 Da, and maximum missed cleavage sites of three. Only the spectrum peaks with a signal-to-noise ratio (S/N) > 4 were selected for searches. The false discovery rate (FDR) was set to 1% at both PSM and peptide levels. The S/N values of both unique and razor peptides were used for protein abundance quantification in each of TMT6 reporter channels. The isotopic impurity correction of the reporter ions was set according to the values provided in accompanying product sheet. To ensure the accuracy of TMT quantification, reporter S/N threshold was set at 10 and co-isolation threshold at 30%. The Functional Enrichment Analysis Tool (FunRich) [19] version 3.1.3 was used for its quantitative gene enrichment analysis in cellular component, biological process, and pathway as previously described [20].

For transcriptomics analysis, the NK gene signature and HIF1A expression were obtained from The Cancer Genome Atlas (TCGA) database on renal cancer and kidney clear cell carcinoma (KIRC). The following genes were used to define the NK cell gene signature: Natural Cytotoxicity Receptor 1 (NCR1), NCR3, Killer Cell Lectin-Like Receptor B1 (KLRB1), CD160, and Perforin 1 (PRF1). Each gene was ranked by its mean expression value [21]. A low NK cell gene signature (NK GS) was defined, if all five genes were lower than their mean values, whereas a high NK GS was defined, if all five genes were higher than their mean values. The expression of HIF1A in both low and high NK GS groups was analyzed using an unpaired *t*-test.

### Multiplex analysis of cytokines and chemokines

Soluble factors in supernatants from ccRCC spheroids or spheroids-NK co-culture were quantified using a 48-parameter Bio-Plex Pro Human Cytokine Screening Panel (Bio-Rad, Hercules, California, USA) on a Bio-Plex 200 Systems instrument (Bio-Rad) according to the manufacturers’ instructions.

### Western blot

Cells were lysed in RIPA buffer (Thermo Scientific) and centrifuged at 12,000 RCF at 4 °C for 10 min. The obtained lysates were mixed with SDS sample buffer (Novex, Waltham, Massachusetts, USA) containing 10% sample reducing agent (Novex). The proteins were resolved by polyacrylamide Bis–Tris 4–12% gradient precast gel (Novex) electrophoresis, followed by transfer onto nitrocellulose membranes (Whatman). After blocking with 5% non-fat milk powder in DPBS with 0.1% tween-20 (Sigma-Aldrich) for 1 h, the membranes were incubated with primary antibodies against HIF1α (NB-100-479; Novus, Burlington, Massachusetts, USA), HIF2α (D9E3) (#7096; CST), VHL (S3-647, 564,183, BD), and GAPDH (ab181602, Abcam, Burlington, Massachusetts, USA). The membranes were then incubated with the corresponding secondary antibodies for 1 h and detected with chemiluminescence (Amersham). The gel images were captured using the iBright CL1500 Imaging System (Invitrogen, Waltham, Massachusetts, USA).

### Opal™ immunostaining and digital image analysis

Formalin-fixed, paraffin-embedded 4 μm sections of tumor spheroids were utilized for immunostaining by Opal™ multiplex reagents (Akoya Biosciences, San Francisco, California, USA). Deparaffinization and rehydration (Decloaking NxGen Chamber BioCare Medical) were performed before the heat-induced epitope retrieval (Citrate Buffer pH 6.0, C9999, Sigma-Aldrich) at 110 °C for 5 min. A 10-min incubation blocking step (Blocking diluent, ARD1001EA, Akoya Biosciences) was performed followed by primary anti-CD45 antibody (1:200 dilution, Abcam) incubation. Samples were incubated for 10 min at room temperature with secondary antibodies (Opal690 anti-Ms HRP, Akoya Biosciences). Opal fluorophores were diluted in amplification diluent (FP1498, Akoya Biosciences) at 1:150 followed by an incubation for 10 min at room temperature in the dark. After the staining, a heat-induced epitope retrieval (Citrate Buffer pH 6.0, Sigma-Aldrich) was performed for 20 min at 95 °C. Slides were mounted with ProLongTM Glass Antifade Mountant with NucBlueTM stain (Thermo Fisher).

Imaging was performed using the Vectra Polaris scanning system (Akoya Biosciences) at 40 × magnification. The QuPath software (0.3.2) was used for cell segmentation and classification. Cell detection was performed using the default settings in QuPath: Threshold: 2, requested pixel size: 0.227 μm, background radius: 8 μm, median filter: 0 μm, and cell expansion: 2 μm. Subsequently, a single classifier for each channel was set up by manual adjustments according to the training area, and a combined classifier was established. The combined classifier was applied to all images, and the results were exported into a.txt file for statistical analysis.

### RCC kidney hybrid organoid culture

Kidney organoid cultures were adapted from a previously published method [22]. Briefly, kidney tissues were cut into small pieces and rinsed with cold DMEM/F12 (Gibco) followed by the generation of kidney cell suspensions using the human Tumor Dissociation Kit (Miltenyi Biotec) protocol. The suspension was filtered through a 100 μm Nylon cell strainer and centrifuged for 4 min at 500RCF. The cell pellet was washed with cold Advanced DMEM/F12 (Gibco, Waltham, Massachusetts, USA) and centrifuged at 500RCF for 4 min. Cells were seeded to a ultra-low attachment 24-well plate (Corning, Corning, New York, USA) and cultured for 3–5 days to self-aggregate into cell clusters. Cell clusters were then collected and cultured in growth factor reduced Matrigel (Corning). Organoids were passaged every 15–20 days in Advanced DMEM/F12 supplemented with penicillin/streptomycin (1%, Gibco), HEPES (1 mM, Gibco), GlutaMAX (1%, Gibco), N-acetylcysteine (1 mM, Sigma) and B27 supplement (2%, Gibco), A83-01 (5 µM, Tocris Bioscience, Waltham, Massachusetts, USA), SB202190 (10 μM, Sigma), Gastrin (10 nM, Sigma), EGF (50 ng/ml, Peprotech), FGF-10 (100 ng/ml, Peprotech), R-Spondin 1 (100 ng/ml, R&D SYSTEMS, Minneapolis, Minnesota, USA), Noggin (100 ng/ml, Peprotech), wnt3a (100 ng/ml, Peprotech), primocine (0.1 mg ml–1, Invivogen, San Diego, California USA), and Rho-kinase inhibitor Y-27632 (10 µM, STEMCELL Technologies, Vancouver, Canada). Organoids with similar size (diameter range from 400 to 500 µm) were selected and cultured with 2000 786-O-GFP or 786-O-pVHL-GFP cells in ultra-low attachment 96 U-bottom plates for 7 days. Four days activated NK cells (100 IU/ml IL-2) were labeled with Dil (1 µg/ml, Thermo Fisher) and then added to RCC kidney hybrid organoids and cultured in Advanced DMEM/F12 supplemented with FBS (10%, Gibco), penicillin/streptomycin (1%, Gibco), HEPES (1 mM, Gibco), GlutaMAX (1%, Gibco), IL-2 (600 IU/ml, Peprotech), and IL-15 (600 IU/ml, Peprotech). Hybrid organoid formation and NK infiltration were monitored by IncuCyte imaging and confocal microscopy (ZEISS, Oberkochen, Germany).

### Statistical analyses

Experimental replicates are presented as mean ± standard deviation (SD) and median in box plot stated in the figure legend of the result section. Statistical analyses were performed using Prism 9 (GraphPad Software) and stated in figure legends as *p < 0.05, **p < 0.01, ***p < 0.001, and **** *p* < 0.0001.

## Results

### VHL restoration in ccRCC confers improved NK cell infiltration

**Fig. 1:**
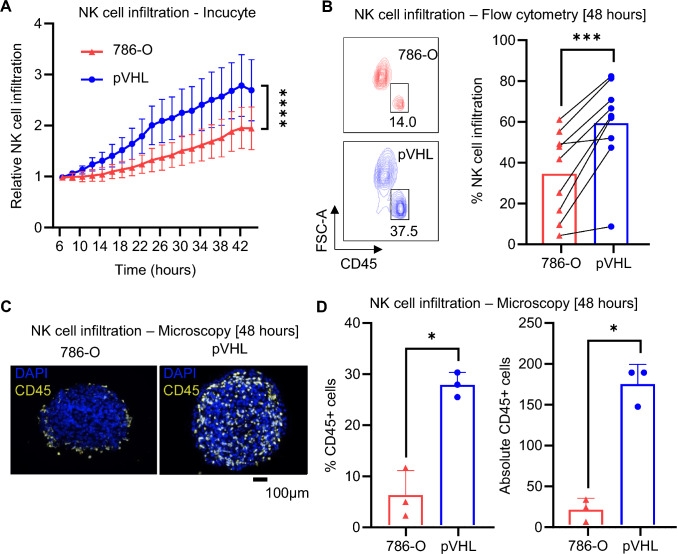
Infiltration of NK cells into ccRCC spheroids. **A** NK cells were added to tumor spheroids at timepoint 0. Cells were left to sediment for 6 h and relative mean NK cell infiltration (by fluorescence) within spheroid growth area was normalized to first reading at 6 h (*n* = 11, mean ± SD). **B** LEFT: representative flow cytometry plots showing CD45-positive NK cell infiltration. The majority (> 94%) of CD45-positive cells were also positive for CD56. RIGHT: NK cell infiltration into spheroids measured by flow cytometer at 48 h after NK cell seeding (*n* = 9). **C** Representative images of paraffin-embedded spheroid slices with Opal™ immunostaining for CD45 and DAPI for nuclei at 48 h. **D** Frequency and number of CD45-positive NK cells in spheroids at 48 h (*n* = 3). Statistical analysis was performed by two-way ANOVA with Dunnett’s multiple comparisons for (**A**) and paired *t*-test for (**B** and **D**) with *p < 0.05, ***p < 0.001, and ****p < 0.0001

Since NK cells have a prognostic value in ccRCC, we investigated if VHL mutations in ccRCC influence NK cell infiltration and activity. When cultured as spheroids, VHL-restored 786-O RCC cells (pVHL) showed a reduced hypoxic status, accompanied by higher VHL expression levels, compared to 786-O RCC with an inherent VHL mutation (Fig. S1C–F). Real-time imaging showed a significantly higher NK cell infiltration in pVHL compared with 786-O spheroids (Fig. 1A and S1G), which was confirmed by flow cytometry analyses with a mean 1.7-fold increased NK cell infiltration in pVHL tumors (Fig. 1B). These results were, furthermore, corroborated by fluorescence imaging demonstrating a significantly higher abundance of NK cells in pVHL compared with 786-O spheroids (Fig. 1C and D). Together, these results show that restoration of VHL renders RCC tumor spheroids more susceptible to NK cell infiltration.

### pVHL-infiltrating NK cells display activated and mature phenotype and increased functional activity

**Fig. 2:**
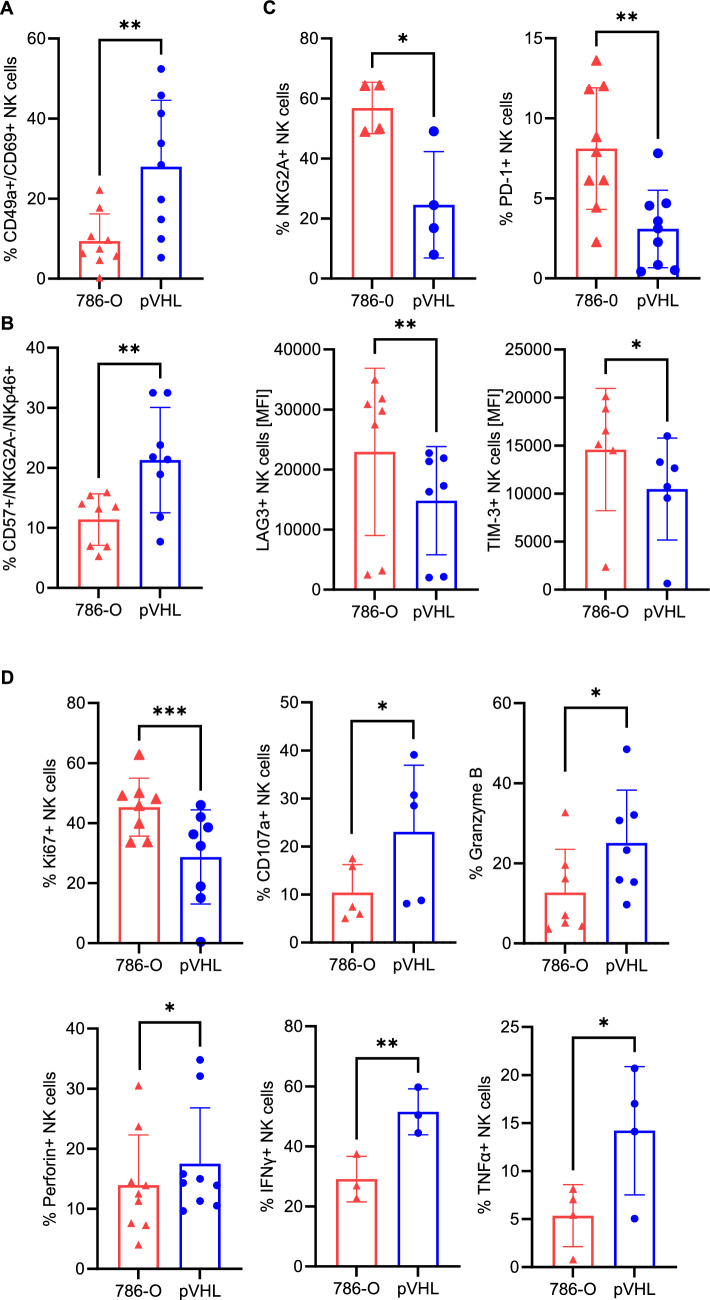
pVHL-infiltrating NK cells display a mature and activated phenotype. **A** Frequency of CD49a + /CD69 + tumor-infiltrating NK cells (48 h, *n* = 9). **B** Frequency of CD57 + /NKG2A-/NKp46 + tumor-infiltrating NK cells (48 h, *n* = 8). **C** Frequency of NKG2A-positive (*n* = 4) and PD-1-positive NK cells (48 h, *n* = 9), and expression of LAG3 (48 h, *n* = 7), and TIM-3 (48 h, *n* = 6) on tumor-infiltrating NK cells. **D** Frequency of tumor-infiltrating NK cells expressing Ki67 (48 h, *n* = 8), CD107a (6–8 h, *n* = 5), Granzyme B (24 h, *n* = 7), perforin (24 h, *n* = 9), IFNy (24 h, *n* = 3), or TNFα (*n* = 4). Each symbol represents a separate biological experiment with NK cells isolated from a different healthy donor. Statistical analysis was performed by paired t-test with *p < 0.05, **p < 0.01, and ***p < 0.001

To investigate potential differences in anti-tumor activity, tumor-infiltrating NK cells were analyzed for their phenotype and function by flow cytometry. Compared with 786-O spheroids, pVHL spheroids were enriched for tissue resident markers CD49a and CD69 double-positive NK cells (Fig. 2A). Furthermore, a higher frequency of mature CD57 + /NKG2A-/NKp46 + NK cells was observed in pVHL compared to 786-O spheroids (Fig. 2B). With regard to immune checkpoint receptors, the frequency of NKG2A and PD-1-positive NK cells was reduced in pVHL spheroids compared to 786-O spheroids. In addition, pVHL-infiltrating NK cells expressed significantly lower levels of LAG-3 and TIM-3 (Fig. 2C). Functionally, pVHL-infiltrating NK cells displayed a significantly reduced proliferation, but increased CD107, IFNγ, TNFα, granzyme B, and perforin levels compared to 786-O infiltrating NK cells (Fig. 2D). Together, these results show that NK cells infiltrating into VHL-restored tumors exhibited a more activated phenotype and function.

### VHL restoration sensitizes ccRCC tumors to NK cell-mediated killing

**Fig. 3:**
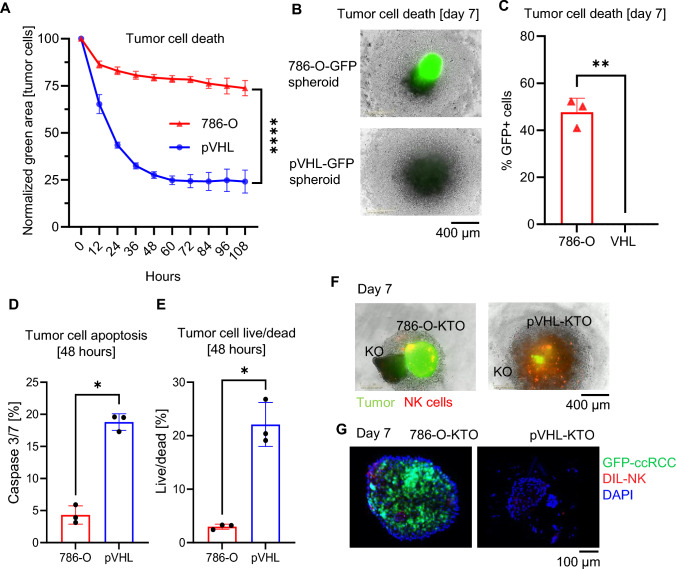
VHL restoration sensitizes ccRCC to NK cell-mediated killing. **A** Relative mean spheroid GFP area normalized to 0 h (*n* = 3, mean ± SD). **B** Representative real-time imaging of NK cells killing RCC-GFP spheroids on day 7. **C** Flow cytometry analysis showing viable (live/dead) GFP-positive RCC tumor cells after 7 days of NK-RCC spheroids co-culture. **D** Frequency of Caspase 3/7-positive tumor spheroids after 48 h of co-culture with NK cells. **E** Frequency of live/dead positive tumor spheroids after 48 h of co-culture with NK cells. **F** Representative real-time imaging of red DIL-labeled NK cells cultured with GFP-positive kidney tumor hybrid organoids (KTO) and kidney organoid (KO) at day 7 of culture. NK cells were labeled with red DIL. Tumor cells are GFP positive. **G** Confocal imaging of 786-O-KTO and pVHL-KTO. Statistical analysis was performed by two-way ANOVA with Dunnett’s multiple comparisons for (A) and paired t-test for (C) with **p < 0.01 and ****p < 0.0001

Given that pVHL spheroids harbor a higher frequency and more activated NK cells than 786-O spheroids, the ability of NK cells to target these tumors was investigated. For these experiments, GFP-positive ccRCC cell lines were generated. While tumor spheroids cultured alone remained viable at > 94%, real-time imaging analysis revealed a significant reduction in GFP-positive pVHL tumor cells compared to GFP-positive 786-O tumor spheroids upon culture with NK cells (Fig. 3A, B and S2A–C). Flow cytometry analyses further confirmed the elimination of pVHL spheroids, whereas 47.6 ± 6.0% 786-O spheroids remained alive (Fig. 3C). In addition, significantly higher frequencies of caspase-3/7 and dead pVHL tumor spheroids were observed upon culture with NK cells (Fig. 3D and E). To substantiate these findings, a tumor kidney hybrid organoid model was developed, where kidney organoids were cultured with or without 786-O or pVHL tumor cells. Similar to the spheroid model, NK cells efficiently killed pVHL tumors, whereas 786-O tumor organoids remained alive. Notably, NK cells infiltrated kidney organoids in the absence of tumor cells, but without signs of destruction of the organoid (Fig. 3F, G, and S2D–E). Thus, the restoration of VHL renders RCC tumor cells more susceptible to NK cell-mediated killing.

### VHL restoration reduces ccRCC progression and immunosuppressive pathways

To investigate underlying mechanisms of increased NK cell infiltration and activation in VHL-restored tumors, quantitative proteomic analysis of pVHL and 786-O spheroids was performed. Several proteins known to play a role in the progression of ccRCC including dipeptidyl-peptidase 4 lymphocyte cell surface protein CD26 (DPP4) [23], collagen type VI alpha 3 chain (COL63) [24, 25], oxidation resistance 1 (OXR1) [26], superoxide dismutase 2 (SOD2) [27], and collagen triple helix repeat containing 1 (CTHRC1) [28, 29] were expressed at significantly higher levels in 786-O spheroids compared to pVHL spheroids. In contrast, the immunomodulating protein S100 calcium-binding protein A2 (S100A2) [30, 31] was significantly higher expressed in pVHL spheroids (Figs. S3A, S4A and Table 1).

**Table 1:** Selected proteins identified in Figure S3A as significantly differentially expressed between pVHL and 786-O tumor spheroids

Gene name	Description	Log (fold change)	Significance in ccRCC and immunomodulation
S100A2	S100 calcium-binding protein A2	1.29	• Interacts with S100A4 and increases NFkB induction and TNFa production in melanoma [30] • Decreased expression in RCC and correlated with p53 mRNA in ccRCC [31]
STX5	Syntaxin-5	1.11	
DPP4	Dipeptidyl-peptidase 4 lymphocyte cell surface protein CD26	−1	• Co-expressed with cancer stemness-related genes in patient-derived RCC spheroid cultures [23]
COL6A3	Collagen type VI alpha 3 chain	−1.46	• Overexpressed in metastatic RCC and associated with poor survival [24] • mRNA 16 × higher in metastatic CAKI-1 than primary CAKI-2 [25]
OXR1	Oxidation resistance 1	−1.74	• Higher OXR1 promoter methylation associated with ccRCC nuclear grade – tumor aggressiveness [26]
SOD2	Superoxide dismutase 2	−1.81	• Involved in oxidative stress and regulated by NUDT1 which is controlled by HIF2a in ccRCC (786-O) [27]
CTHRC1	Collagen triple helix repeat containing 1	−2.20	• Upregulated in RCC tissues and cell lines, involved in proliferation, EMT migration and invasion in ccRCC (CAKI-1) [28] • High CTHRC1 predicted worse prognosis in KIRC based on immune cells [29]

To elucidate the immunomodulatory role of VHL restoration, quantitative gene ontology (qGO) of the identified proteins and their corresponding abundances was performed. Proteins related to cellular components involved in DNA replication along with NFkB signaling were higher, whereas extracellular matrix (ECM), secretory, and antigen processing components were lower in VHL-restored tumors (Fig. S4B). Furthermore, biological process qGO analysis revealed lower cell proliferation, growth, and motility in VHL-restored tumors (Fig. S4C). Upon examining specific biological pathways, VHL-restored tumors showed lower expression of MHC class I processing and presentation and PD-1 signaling (Fig. S3B). In addition, specific protein abundance of the immunomodulatory proteins TIMP1 and Galectin-1 were lower in VHL-restored tumors compared to VHL-mutated tumor spheroids (Fig. S4D). Taken together, these results showed several changes related to tumor progression and immunomodulatory pathways in ccRCC upon VHL restoration.

### Altered secretome in pVHL and 786-O tumor spheroids

Given that several changes related to tumor progression and immunomodulatory pathways in ccRCC upon VHL restoration were observed, secretome analysis of pVHL and 786-O spheroids was performed to investigate additional underlying mechanisms of increased NK cell infiltration in VHL-restored tumors. Multiple cytokines were present at higher levels in 786-O spheroids compared to pVHL spheroids. In line with our quantitative proteomics analysis, the CXCR2 ligands GROα and IL-8 were the highest secreted factors by 786-O tumor spheroids (Fig. S3C).

When cultured with NK cells, the levels of several soluble factors differed between cultures with 786-O and pVHL spheroids (Fig. S5). Despite a high donor variability, several soluble factors showed consistent and significant differences between 786-O and pVHL spheroids. In line with our flow cytometric data, higher IFNγ levels were observed in co-cultures with pVHL spheroids. In addition, levels of IL-9, PDGF-bb, and MCP-1 were higher in co-cultures with pVHL spheroids whereas IL-10, TRAIL, VEGF, and SCF were higher in co-cultures with 786-O spheroids (Fig. S3D). Together, these results reveal several changes in the secretome between VHL-mutated and -restored RCC tumors, and that CXCR2 ligands may influence infiltration the NK cell infiltration into VHL-restored tumor spheroids.

### Inhibition of HIFα increases NK cell infiltration into 786-O tumor spheroids

Although differences in specific biological pathways related to MHC class I processing and presentation and β2 integrin cell surface interaction (Fig. S3), and abundance of HLA-A and ICAM-1 was observed between 786-O tumors compared to pVHL tumors, blockade of MHC class I and ICAM-1 did not influence the NK cell infiltration into 786-O tumors (Fig. S6A and S6B). Although hypoxia modulates the immunosuppressive factors COX-2 and IL-10 in the TME [32, 33], and IL-10 was detected at higher levels in cultures with NK cells and 786-O spheroids, neutralization of COX-2 and IL-10 did not influence NK cell infiltration into 786-O tumors (Fig. S6C and S6D). Since CXCR2 ligands were higher in 786-O spheroids compared to pVHL spheroids, neutralization of IL-8 did not have an impact on NK cell infiltration into 786-O tumors (Fig. S6E).

**Fig. 4:**
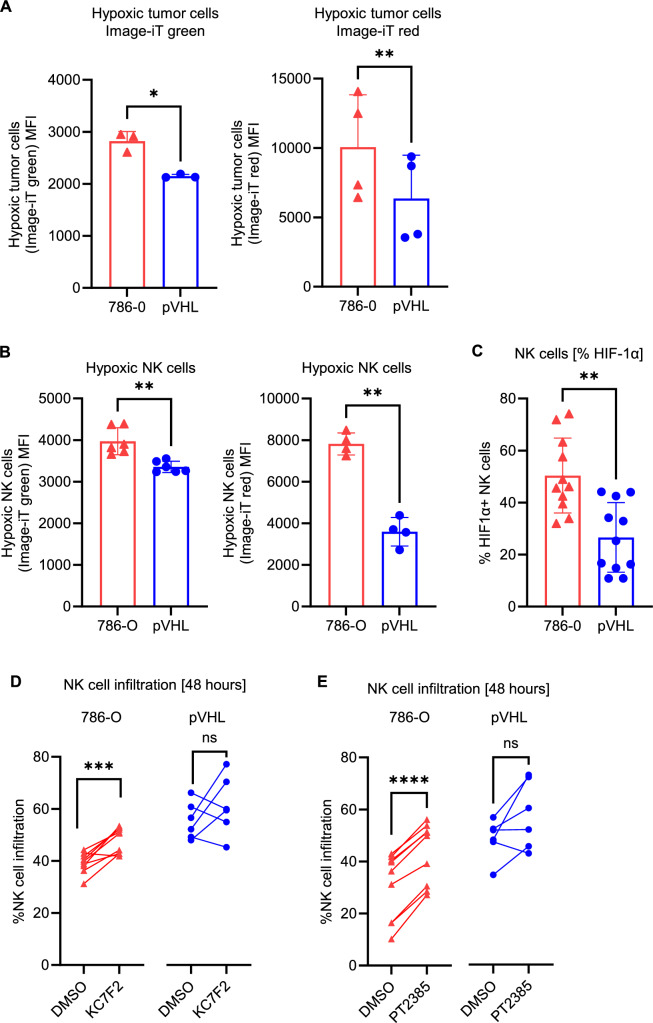
Inhibition of HIFα increases NK infiltration. **A** Flow cytometric analysis of hypoxic status of NK cells (CD45 +) using Image-iT green fluorescence (*n* = 3, LEFT) and red fluorescence (*n* = 4, RIGHT) reagents in tumor spheroids. **B** Flow cytometry analysis of hypoxic status in NK cells (CD45 +) using Image-iT green fluorescence (*n* = 6, LEFT) reagents and red fluorescence (*n* = 4, RIGHT) in tumor-infiltrating NK cells. **C** Frequency of HIF-1α-positive tumor-infiltrating NK cells (CD45 + , *n* = 11). **D** NK cell infiltration upon inhibition of HIF-1α (KC7F2). KC7F2 was added to NK cells for 48 h and then washed prior to culture with tumor spheroids (*n* = 9). **E** NK cell infiltration upon inhibition of HIF-2α (PT2385). PT2385 was added to tumor cells on day 0 during the formation of spheroids (*n* = 6). Viability (Caspase 3/7-negative cells) of tumor spheroids remained above 90% after treatment with 10 µM PT2385. Statistical analysis was performed by paired *t*-test with *p < 0.05, **p < 0.01, ***p < 0.001, ****p < 0.0001, and ns = non-significant

Since HIF1α expression has been shown to interfere with NK cell function [34], the role of HIF1α in NK cells was investigated in relation to the ability to infiltrate tumor spheroids. When cultured with NK cells, both pVHL spheroids and pVHL-infiltrating NK cells showed reduced hypoxia status compared with 786-O spheroids and 786-O-infiltrating NK cells (Fig. 4A and B). Furthermore, pVHL-infiltrating NK cells showed lower expression of HIF1α compared with 786-O infiltrating NK cells (Fig. 4C). Upon inhibition of HIF1α in NK cells, increased NK cell infiltration was observed in 786-O, but not in pVHL spheroids (Fig. 4D). Since HIF2α expression in RCC tumors has been shown to regulate NK cell activity [35], inhibition of HIF2α was investigated in relation to NK cell infiltration. Upon inhibition of HIF2α in spheroid cultures, NK cell infiltration was significantly higher into 786-O tumors compared with pVHL spheroids (Fig. 4E). Together, these results show that the inhibition of MHC class I, ICAM-1, COX-2, IL-10, or IL-8 does not influence NK cell infiltration, whereas inhibition of either HIF1α or HIF2α enhances NK cell infiltration into VHL-mutated tumors.

## Discussion

In human ccRCC, VHL is the most commonly mutated gene that drives the development of the disease [36]. Since NK cells play an important role in immune surveillance of ccRCC [6–8], it is important to understand how the mutational status of VHL influences their activity. Here, we investigated the effects of VHL status on NK infiltration, phenotype, and function and found that restoration of VHL in ccRCC improves NK cell infiltration and activity. In pVHL spheroids, the improved NK cell infiltration was accompanied by a significantly higher proportion of NK cells expressing CD49a and CD69. CD49a has been used to define kidney tissue resident NK cells, where it associates with CD69 and S1PR1 leading to their retention within tissues [37]. Moreover, NK cells infiltrating pVHL spheroids displayed a more mature phenotype characterized by the expression of CD57, NKp46, and the absence of NKG2A. The expression of these NK cell receptors has previously been shown to be modulated by the ccRCC TME [38]. Accordingly, these mature NK cells showed a reduced proliferation suggesting that the increase in NK cell frequency in VHL-restored tumors is caused by infiltration rather than proliferation. The higher expression of inhibitory immune checkpoint receptors including PD1, LAG3, and TIM3 on NK cells infiltrating VHL-mutated tumors might indicate an inferior functional capacity of these NK cells. Although VHL-mutated tumors harbored reduced frequencies of NK cell expressing CD107a, granzyme B, perforin, IFNγ, and TNFα compared with VHL-restored tumors, further investigations are needed to comprehensively study how the expression of inhibitory immune checkpoint receptors are linked to functional activity in VHL-mutated versus -restored tumors.

Multiple factors known to be involved in tumor progression and regulation of immunity including MHC class I, PD-L1, IL-8, IL-10, and COX-2 were differentially expressed between 786-O and pVHL spheroids. However, neutralization of these factors did not impact on the infiltration of NK cells. Since hypoxia can influence the expression of adhesion molecules, the mutational status of VHL may influence the physical properties of tumor spheroids. Although we observed increased ICAM-1 expression in VHL-mutated tumor spheroids, neutralization of ICAM-1 did not impact on the infiltration of NK cells. Nevertheless, it could be of interest to explore if physical properties of tumor spheroids including spheroid size and cellular density influence NK cell infiltration.

A higher frequency of HIF1α-positive tumor-infiltrating NK cells were observed in VHL mutant RCC tumor spheroids compared with VHL-restored RCC tumor spheroids. Upon inhibition of HIF1α in NK cells, an increased infiltration was observed in VHL mutant 786-O RCC tumor spheroids. These results agree with the previous studies where HIF1α is associated with reduced function of tumor-infiltrating NK cells, and deletion of HIF1α in NK cells reduces tumor progression [34, 39]. Similar to HIF1α, accumulation of HIF2α results in overexpression of inositol triphosphate receptor-1 (ITPR1), thereby protecting 786-O RCC cells from NK cell-induced autophagy [35]. Upon inhibition of HIF2α, a greater infiltration was observed in VHL mutant 786-O RCC tumor spheroids. It would be of potential interest to understand how HIF2alpha changes the tumor proteome to influence NK cell infiltration, preferentially coupled with studies using organoid models to investigate NK cell infiltration from normal kidney tissue compartments into the tumor compartment. Still, inhibition of HIFα might represent a promising therapeutic strategy for patients with ccRCC. For example, strategies to activate NK cells along with treatment with the recently approved HIF2α inhibitor, belzutifan, could be explored [40].

## Conclusions

By utilizing unique tumor spheroid models of RCC to better recapitulate a 3D growing tumor, we demonstrate that VHL-mutated tumors are less infiltrated by NK cells compared with VHL-restored tumors. Furthermore, NK cells in VHL-restored tumors showed an activated phenotype with the ability to reduce tumor spheroid size. Proteomic analysis revealed a higher expression of MHC class I, ICAM-1, COX-2, IL-8, and IL-10 in VHL-mutated tumors. However, inhibition of these factors had no impact on NK cell infiltration. Instead, inhibition of HIF1α and HIF2α resulted in higher NK cell infiltration into VHL-mutated tumors. Thus, inhibition of HIFα represents a valid strategy to improve NK cell anti-tumor efficacy against VHL-mutated tumors.

## Supplementary Information

Below is the link to the electronic supplementary material.

Supplementary file1 (PDF 863 kb)

## Data Availability

No datasets were generated or analysed during the current study.

## Abbreviations

3D: Three-dimensional
Ab: Antibody
ccRCC: Clear cell renal cell carcinoma
COL63: Collagen Type VI Alpha 3 Chain
COX-2: Cyclooxygenase-2
CTHRC1: Collagen triple helix repeat containing 1
DPP4: Dipeptidyl-peptidase 4 lymphocyte cell surface protein CD26
ECM: Extracellular matrix
FBS: Fetal bovine serum
FC: Flow cytometer
HIF: Hypoxia-inducible factor
HLA: Human leukocyte antigen
IFNγ: Interferon-gamma
IL-15: Interleukin-15
ITPR1: Inositol triphosphate receptor 1
KIRC: Kidney clear cell carcinoma
Lag-3: Lymphocyte-activation gene 3
MHC: Major histocompatibility complex
NF-κB: Nuclear factor kappa-light-chain-enhancer of activated B cells
NK: Natural killer cells
OXR1: Oxidation resistance 1
PBMC: Peripheral blood mononuclear cells
PD-1: Programmed cell death protein 1
PD-L1: Programmed death-ligand 1
PGE-2: Prostaglandin E2
qGO: Quantitative gene ontology
RCC: Renal cell carcinoma
ROS: Reactive oxygen species
S100A2: S100 calcium-binding protein A2
SOD2: Superoxide dismutase 2
TCGA: The Cancer Genome Atlas
TIM-3: T-cell immunoglobulin and mucin-domain containing-3
TME: Tumor microenvironment
TNFα: Tumor necrosis factor
VHL: Von Hippel–Lindau
