# Supplementary material for: VHL restoration in clear cell renal cell carcinoma improves NK cell infiltration and function
Source: Cancer Immunol Immunother. 2025 Jul 30;74(9):278. doi: 10.1007/s00262-025-04132-x (PMC12311085; doi:10.1007/s00262-025-04132-x)
Supplement: Supplementary file 1 — Supplementary file1 (PDF 863 kb) [file 262_2025_4132_MOESM1_ESM.pdf]

Supplementary Files for

VHL restoration in clear cell renal cell carcinoma improves NK cell infiltration and function

Le Tong†[1,2], Apple Hui Min Tay†[1,3], Weiyingqi Cui[1], Yaxuan Liu[1], Yanhong Su[1], Jiawen Lyu[1], Leila Hoedemakers[1], Ying Yang[1], Monika Ehnman[1], Barbara Seliger[4,5,6], Par Nordlund[1], Felix Haglund de Flon[1], Shi Yong Neo[1,7], Andreas Lundqvist[1]

[1] Department of Oncology-Pathology, Karolinska Institutet, Stockholm, Sweden.

[2] Key Laboratory of Quantitative Synthetic Biology, Shenzhen Institute of Synthetic Biology, Shenzhen Institute of Advanced Technology, Chinese Academy of Sciences, Shenzhen, China

[3] School of Biological Science, Nanyang Technological University, Singapore, Republic of Singapore.

[4] Faculty of Health Sciences Brandenburg, Institute of Translational Immunology, Medical School "Theodor Fontane", Brandenburg an der Havel, Germany.

[5] Center of Translational Medicine, Medical School "Theodor Fontane", Brandenburg an der Havel, Germany.

[6] Medical Faculty, Martin Luther University of Halle-Wittenberg, Halle (Saale), Germany.

[7] Singapore Immunology Network, Agency for Science, Technology and Research (A*STAR), 8A Biomedical Grove, Singapore 138648, Republic of Singapore

Corresponding author: Andreas Lundqvist, J6:20 BioClinicum, Akademiska straket 1, 17164 Solna, +46-(0)8-524 823 71, andreas.lundqvist@ki.se.

†Equal contribution

Supplemental Fig 1. NK cell infiltration and killing of tumor spheroids. (A) Purity of NK cells after isolation from two representative healthy donors. Cells are gated for single live cells. **(B)** LEFT: expression of CD56 on CD45+ tumor-infiltrating NK cells. MIDDLE: frequency of CD56+ NK cells among CD45+ tumor-infiltrating NK cells from six different NK cell cultures. RGHT: Expression of CD45 on RCC and NK cells. **(C)** Western blot analysis of ccRCC cell lines with inactivated (786-O) and restored VHL (pVHL). **(D)** Protein abundance of VHL in tumor spheroids as measured by LC-MS/MS (*n*=3). **(E)** Representative image of hypoxic status on day five of spheroid culture using Image-iT Red fluorescence reagent. **(F)** Hypoxic status on day five of spheroid culture using Image-iT Green fluorescence reagent (*n*=5). **(G)** Real-time images of spheroid NK cell infiltration over 48 hours under 4x objective. Red areas demarcate CellTracker Deep Red labelled NK cells infiltrating into the spheroid. Unpaired t-tests were performed with $*p < 0.05$.

Supplemental Fig 2. NK cell infiltration into tumor spheroids and organoids. (A) images of GFP positive 786-O and pVHL tumor cells. **(B)** frequency of GFP positive 786-O and pVHL tumor cells. **(C)** GFP intensity of tumor spheroids alone or in co-culture with NK cells. **(D)** Real-time imaging of NK cells killing kidney-tumor hybrid organoids at day 0-6 of NK cell co-culture. **(E)** Images of red DIL-labeled NK cells (highlighted by red arrows) cultured with kidney organoids without tumor cells. Examples of two kidney organoids are shown.

Supplemental Fig 3. Proteomic and secretome analysis of 786-O versus pVHL tumor spheroids. **(A)** Volcano plot of differentially expressed proteins of pVHL and 786-O tumor spheroids (*n*=3 biological replicates). **(B)** FunRich quantitative gene ontology analysis in Biological Pathway. Term “Antigen Presentation: Folding assembly and peptide loading of class IMHC” is abbreviated as “Antigen Presentation” **(C)** Fold change in expression of secreted factors between 786-O and pVHL tumor spheroids (cutoff >1.2-fold change). **(D)** Different levels of cytokines in cultures of NK cells and tumor spheroids. Statistical analysis was performed by paired t tests with $*p < 0.05$, $**p < 0.01$, and $***p < 0.001$.

Supplemental Fig 4. Proteomic analysis of 786-O and pVHL tumor spheroids. (A) Corresponding abundance among the differentially expressed proteins. FunRich quantitative gene ontology analysis in **(B)** Cellular Component and **(C)** Biological Processes. **(D)** Protein abundance of TIMP1 and Galectin-1. Statistical analysis was performed by paired t tests with $*p < 0.05$, $**p < 0.01$ and $***p < 0.001$.

Supplemental Fig 5. Secretome analysis of spheroids in the presence of NK cells. Heatmap showing expression levels of 47 human cytokines in the supernatant of spheroids cultured with three separate NK cell donors for 48 hours (NK1-3).

Supplemental Fig 6. Inhibition of MHC class I, ICAM-1, COX-2, IL-10 or IL-8 does not influence NK cell infiltration. (A) Protein abundance of HLA-A (LEFT) and ICAM-1 (RIGHT) in tumor spheroids by quantitative proteomics analysis (*n*=3 biological replicates). **(B)** Frequency of tumor-infiltrating NK cells upon blockade of MHC class I (*n*=4) or ICAM-1 (*n*=3) at 20 hours. Anti-MHC class I and anti-ICAM-1 antibodies were added to cultures together with NK cells on day 5. **(C)** Expression of COX-2 (LEFT, *n*=8) and IL-10 (RIGHT, *n*=13) in 786-O and pVHL spheroids. **(D)** Frequency of tumor-infiltrating NK cells upon inhibition of COX-2 or neutralization of IL-10 (*n*=4). Inhibitors were added to cultures together with NK cells on day 5. **(E)** Frequency of tumor-infiltrating NK cells upon inhibition of IL-8 added to cultures together with NK cells on day 5 (*n* = 5). Statistical analysis was performed by paired t tests with $*p < 0.05$ and $**p < 0.01$.

Supplementary Figure 1

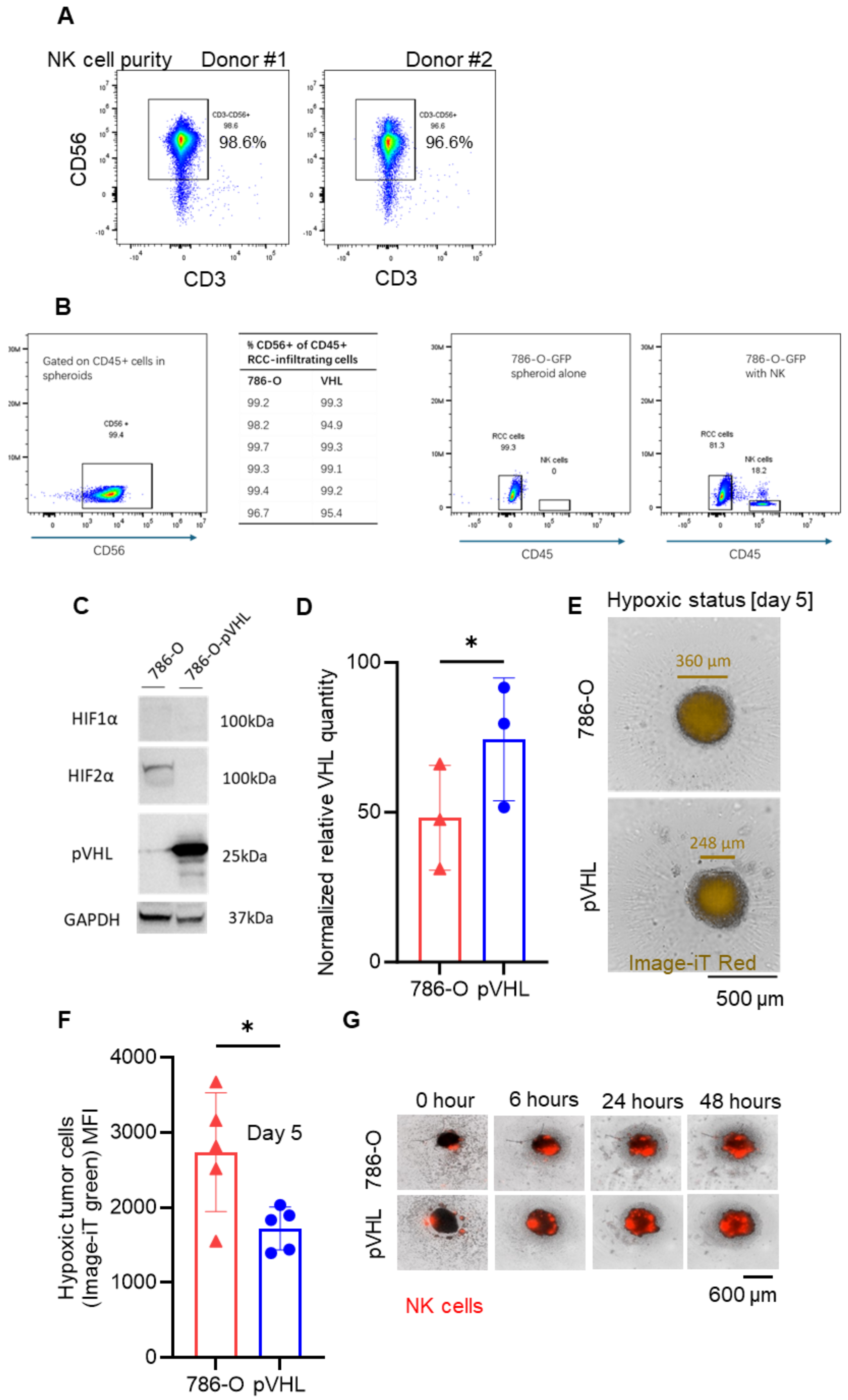

Supplementary Figure 2

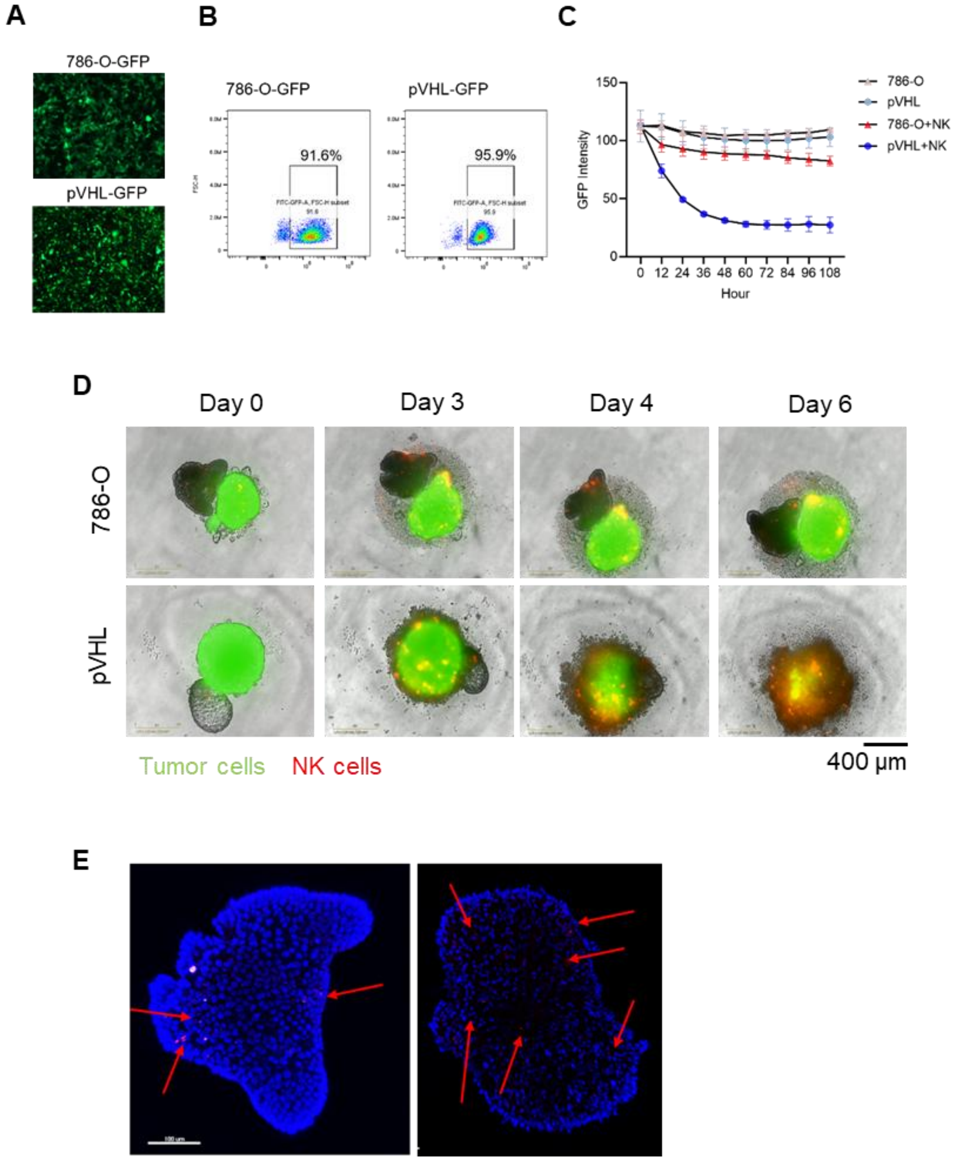

Supplementary Figure 3

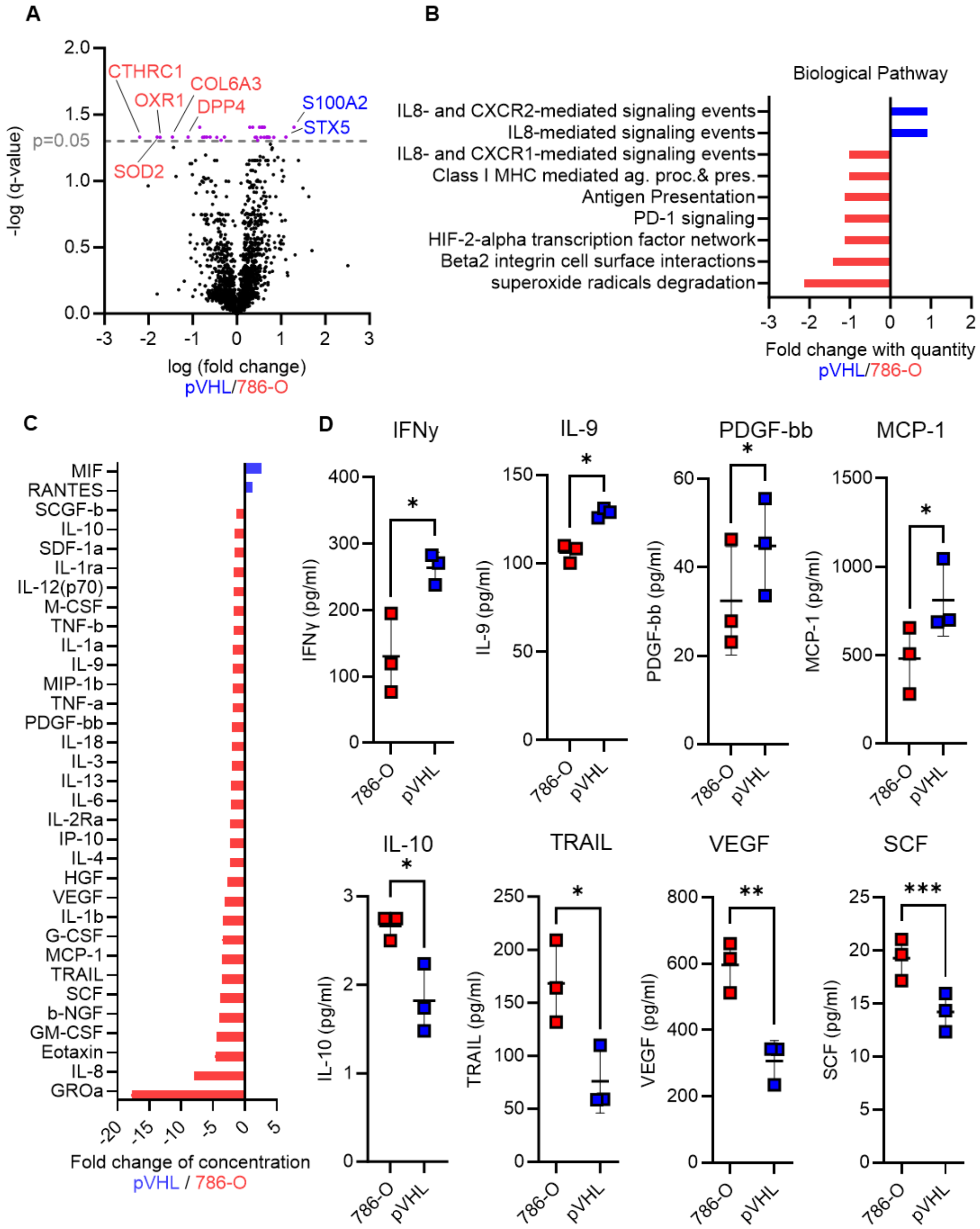

Supplementary Figure 4

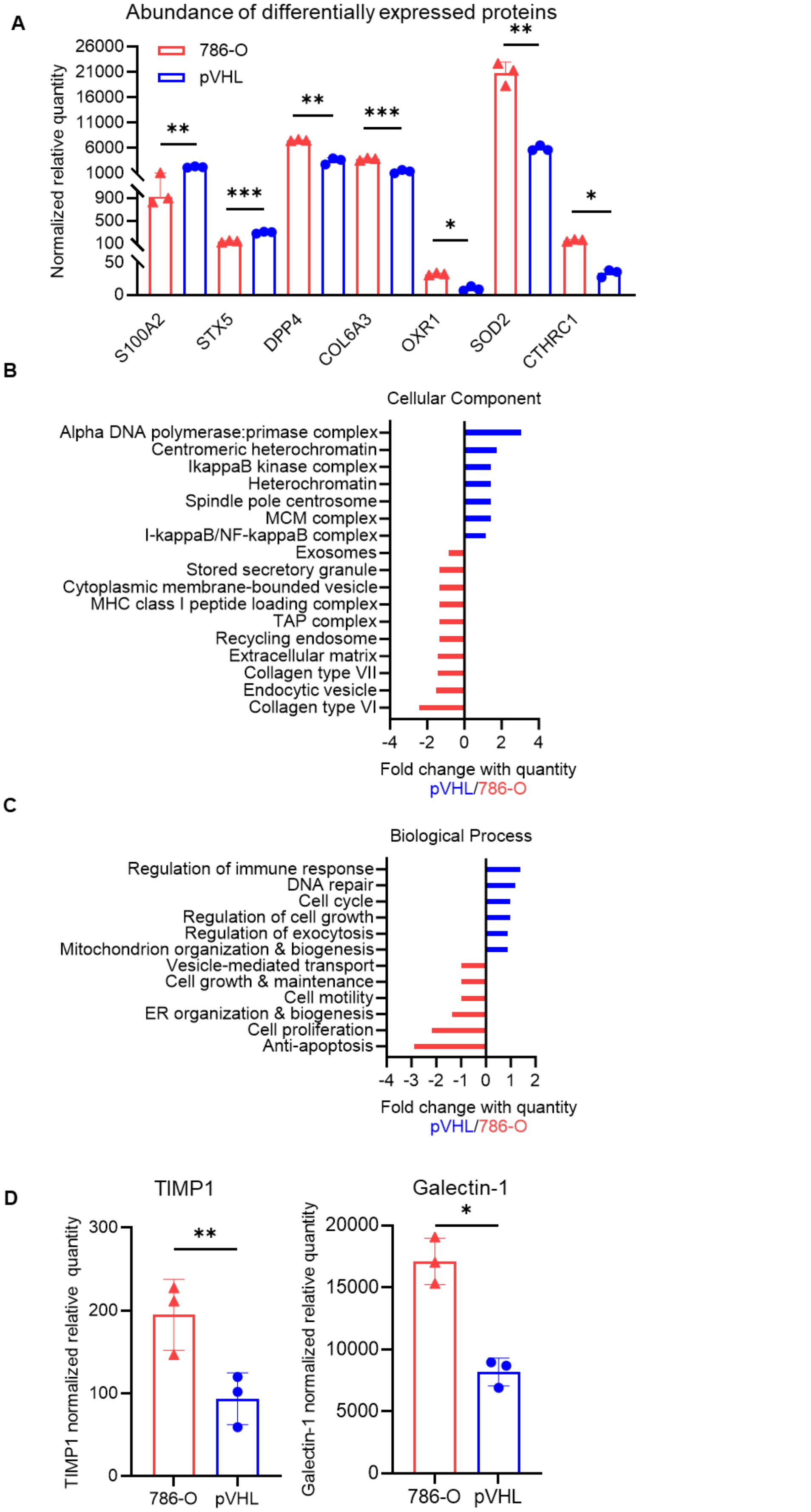

Supplementary Figure 5

Secretome of spheroid/NK cell cultures [48 hours]

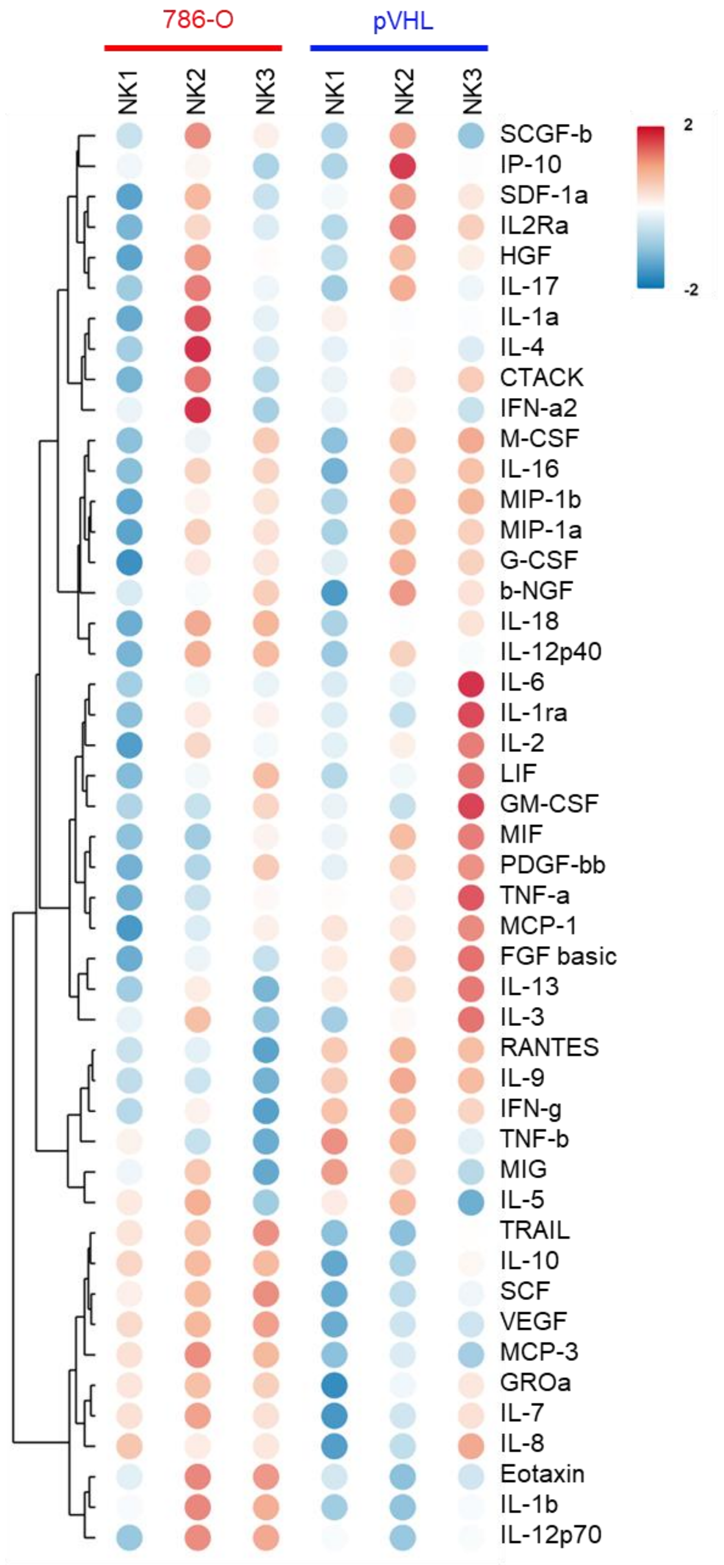

Supplementary Figure 6

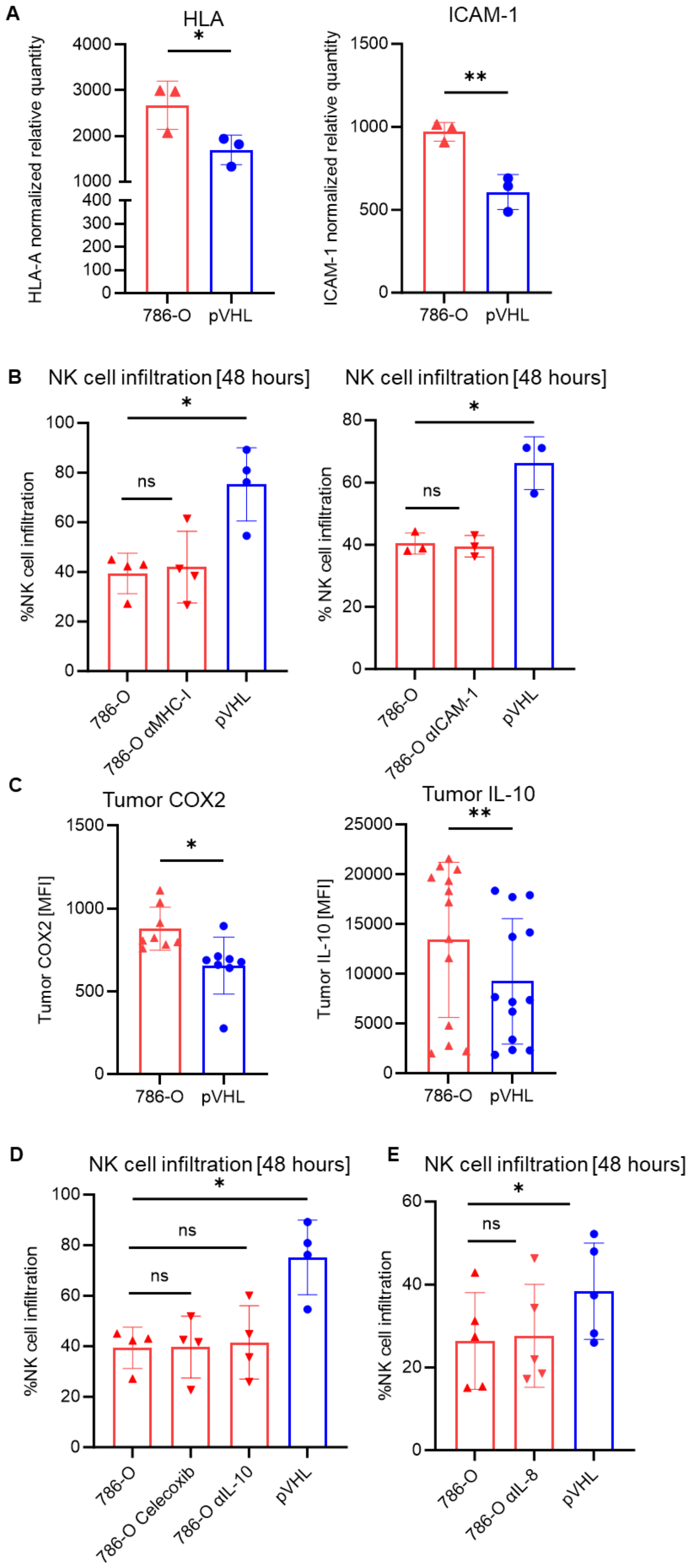

Supplementary Table 1. List of antibodies used for flow cytometry. **A.** NK cell spheroid infiltration. **B.** Degranulation and cytokine production.

Antigen	Conjugate	Vendor	Catalog Number	A	B
CD45	PE-CF594	BD Biosciences	562279	√	√
	BV 650	BioLegend	304044	√	
	PerCP-eFluor 710	Thermo Fisher Scientific	46-0459-42	√	√
CD56	BV 570	BioLegend	318330	√	√
	AF 700	BioLegend	318316	√	
	BV 785	BioLegend	362549	√	
CD57	BV 605	BioLegend	393303	√	
	Pacific Blue	BioLegend	322316	√	
NKG2A	PerCP	RD Systems	FAB1059C	√	
	PE-CY7	Beckman coulter	B10246	√	
NKp46	APC	BD Biosciences	558051	√	
	Pacific Blue	BD Biosciences	562099	√	
	PE-CY7	Thermo Fisher	25-5878-42	√	
Ki67	BV 785	BD Biosciences	563756	√	
HIF1α	PE	BioLegend	359704	√	√
CD69	AF700	BD Biosciences	560739	√	
CD49a	APC-Cy7	BioLegend	328317	√	
HLA-ABC	Spark NIR 685	BioLegend	311451	√	
	FITC	BD Biosciences	560965	√	
PD-L1	BV 785	BioLegend	329736	√	
	PE-CY7	BD Biosciences	558017	√	
PD-1	Pacific Blue	BioLegend	329916	√	
	APC	Thermo Fisher	17-9969-42	√	
COX-2	PE	BD Biosciences	565125	√	
ICAM-1	PE	BioLegend	322708	√	
TIM-3	BV 785	BioLegend	345032	√	
LAG-3	AF-750	BioLegend	369214	√	
IL-10	PE-Cy7	BioLegend	501420	√	
CD107a	PE-Cy5	Thermo Fisher	15-1079-42		√
Perforin	APC/Fire 750	BioLegend	353318		√
Granzyme B	Pacific Blue	BioLegend	515403		√
IFN γ	FITC	BD Biosciences	340449		√
TNFα	BV785	BioLegend	502948		√
Live/Dead marker	Aqua	Thermo Fisher	L34966	√	√
Live/Dead marker	Near-IR	Thermo Fisher	L34975	√	
